# Coherent and dynamic beam splitting based on light storage in cold atoms

**DOI:** 10.1038/srep34279

**Published:** 2016-09-28

**Authors:** Kwang-Kyoon Park, Tian-Ming Zhao, Jong-Chan Lee, Young-Tak Chough, Yoon-Ho Kim

**Affiliations:** 1 Department of Physics, Pohang University of Science and Technology (POSTECH), Pohang 37673, Korea; 2 Department of Medical Technology, Gwangju University, Gwangju 61743, Korea

## Abstract

We demonstrate a coherent and dynamic beam splitter based on light storage in cold atoms. An input weak laser pulse is first stored in a cold atom ensemble via electromagnetically-induced transparency (EIT). A set of counter-propagating control fields, applied at a later time, retrieves the stored pulse into two output spatial modes. The high visibility interference between the two output pulses clearly demonstrates that the beam splitting process is coherent. Furthermore, by manipulating the control lasers, it is possible to dynamically control the storage time, the power splitting ratio, the relative phase, and the optical frequencies of the output pulses. With further improvements, the active beam splitter demonstrated in this work might have applications in photonic photonic quantum information and in all-optical information processing.

Photonic quantum computation has received much attention over the last decade due to the demonstration of scalability using single-photon sources, projective measurement, and linear optical elements such as beam splitters, phase shifters, and mirrors^1^. In addition to these elements, quantum memories are essential in synchronizing multi-photon events to increase the success probability of linear optical quantum gates^1^,^2^,^3^. Although recent advances in miniaturized photonic circuits on a silicon chip may reduce the experimental overhead in managing many such linear optical elements^4^, it does not actually decrease the number of such elements. By employing EIT-based light storage, we demonstrate that a weak laser pulse can be coherently and dynamically manipulated in a cold atom ensemble to perform beam splitting, reflection, and phase shifting, in addition to quantum memory operation. This kind of approach may curtail the number of required elements in that such a unit can function as several optical components and is reconfigurable according to requested operations.

Beam splitters play a crucial role in quantum optics and photonic quantum information, as demonstrated in the Hanbury-Brown–Twiss experiment^5^, the Hong-Ou-Mandel effect^6^, implementation of photonic quantum gates^1^,^4^, etc. While beam splitters based on passive linear optics are widely available at low cost, there are growing needs for dynamic beam splitting devices in which the splitting ratio can be easily varied. Moreover, such devices integrated with coherent optical memory functionalities can significantly reduce resource requirement in photonic quantum information and all-optical information processing. Coherent optical memories or quantum memories for photons have recently been demonstrated by utilizing coherent atom-photon interaction schemes, e.g., EIT^7^,^8^,^9^,^10^, the DLCZ protocol^11^,^12^, and Raman memory^13^,^14^. Dynamic beam splitting devices based on such atom-photon interaction schemes, therefore, would allow an integrated photonic device in which quantum memory and linear optical functionalities are combined.

Recently, controllable beam splitting in frequency or time modes has been demonstrated by using the gradient echo memory scheme^15^, with a double-tripod EIT scheme^16^, and with a tripod EIT scheme^17^. Spatial beam splitting has been demonstrated in warm atomic vapor relying on the EIT effect and natural atomic thermal motions in which atoms with light-induced ground state coherence can move freely to another spatial channel at which the ground state coherence is retrieved back to light^18^. Since this scheme relies on atomic thermal random motion, only a small fraction of the input light can be retrieved at the second spatial channel. There have been several theoretical proposals for efficient spatial splitting of a signal beam by using a cold atom medium with two control beams^19^,^20^,^21^.

In this paper, we propose and demonstrate coherent and dynamic beam splitting of a weak laser pulse into two spatial modes, precisely resembling a variable beam splitting operation of a linear optical device^22^. Our scheme is based on the EIT-based light storage scheme in a rubidium cold atomic ensemble: The photonic excitation of a weak laser pulse is mapped into an atomic coherence, called the spin wave^7^,^10^. After a controlled delay, two nearly counter-propagating control beams are applied to retrieve the signal beam into two spatial modes whose directions are determined by the phase matching condition. As the scheme is based on a phase-matched process, light retrieval can be highly efficient. Experimentally, we demonstrate dynamic beam splitting with the quantum memory operation. We show that, between the two output spatial modes, it is possible to control the power splitting ratio, the relative phase, and the relative frequency shift. Although spatial beam splitting based on a phase-matched process was implemented in solid-state systems^23^,^24^, phase coherence between retrieved beams has not been demonstrated despite its importance in coherent optical information processing. Here, we demonstrate not only the phase coherence between two output beams, but also phase controllability of each output via a phase transfer mechanism from the corresponding control light to the output beam.

## Results

### Schematic and theory

**Figure 1:**
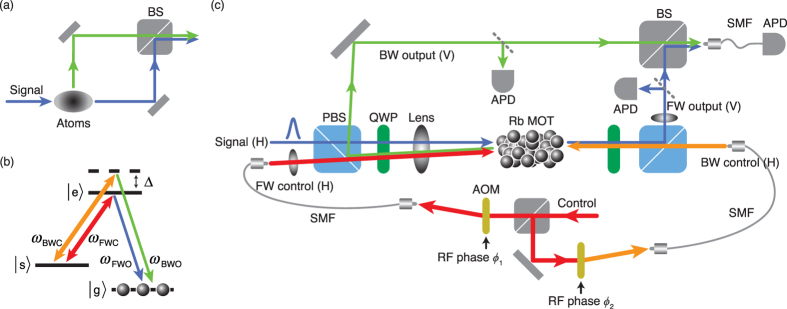
Experimental setup. (**a**) Conceptual schematic of the Mach-Zehnder interferometer utilizing the beam splitter based on EIT light storage in a cold atom ensemble. (**b**) The input signal is resonant with |*g*〉-|*e*〉. Two control lasers are applied between |*s*〉-|*e*〉: on resonant for the forward control (*ω*_FWC_) and with a detuning Δ for the backward control (*ω*_BWC_). The input signal is then split into the two output beams: forward output (*ω*_FWO_, resonant to |*g*〉-|*e*〉) and backward output (*ω*_BWO_, with a detuning Δ to |*g*〉-|*e*〉). All fields are *σ*^+^ polarized. (**c**) The weak signal pulse is launched into an ^87^Rb MOT when the strong forward (FW) control beam is turned on. The angle between the FW control and the signal is 0.3°. Storage of the signal is achieved simply by turning off the FW control. After a certain storage time, the input signal can be retrieved into the forward (FW) or backward (BW) outputs by turning on the FW or BW controls, respectively. AOM: acousto-optic modulator. BS: non-polarizing beamsplitter. PBS: polarizing beamsplitter. QWP: quarter wave plate. SMF: single-mode fiber. APD: avalanche photodiode.

The essential features of the experiment are depicted in Fig. 1(a). A weak Gaussian-shaped signal pulse of ~50 nW is split into two directions at the atomic ensemble after a certain period of coherent light storage. The two output beams are then superposed at a linear optical beam splitter to form a Mach-Zehnder interferometer. The energy level diagram for the cold atoms and the detailed experimental schematic are shown in Fig. 1(b,c), respectively. The horizontally (H) polarized input signal pulse is transformed to right circular polarization and focused into the atomic ensemble of cold atoms of 87 rubidium prepared in a magneto-optical trap (MOT). The forward (FW) control is turned on to open the transparency window for the signal pulse and then adiabatically turned off to map the signal pulse into the atomic spin wave. The angle between the FW control and the signal is 0.3°. After a programmable storage, the controls are turned back on to convert the spin wave back into photonic excitation, i.e., the output beams. The direction 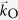 and the frequency *ω*_O_ of the output are determined by the following phase matching condition^25^,


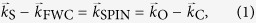



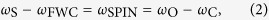


where 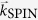 (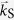, 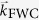) and *ω*_SPIN_ (*ω*_S_, *ω*_FWC_) are the wave vector and frequency of the spin wave (signal, FW control), respectively. The wave vectors and the frequency of the control beams (i.e., retrieval control) used for generating the output beams are 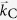 and *ω*_C_, respectively.

According to equation (1), if we assume 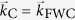, then 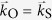, i.e., the direction of the output signal is the same as that of the input if the retrieval control is the same as the FW control. On the other hand, if we consider the condition 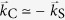, then 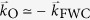. That is, if the retrieval control is in the backward (BW) direction, the output is nearly opposite to the FW control as sketched in Fig. 1(c). The input signal beam can be split into two output directions if both FW and BW controls are applied to the cold atom medium at the same time, thus functioning as a dynamic beam splitter. Note that this process is not possible in a warm vapor. In a medium with a large Doppler shift, instead of beam splitting, light retrieval can occur only in the spatial mode with the stronger control field. This is due to the fact that higher spatial-frequency components of Raman coherence decay quickly in a warm vapor^19^,^26^. Assuming that the magnitudes *k* of the wave vectors of controls and signals are the same, zero-order spatial Raman coherence *ρ*_*sg*_ is formed by the co-propagating controls and signals. The higher order Raman coherences *ρ*_*sg*_*e*^±2*ikz*^ are formed by the counter-propagating controls and signals. The higher order Raman coherences decay faster in atomic warm vapor than in colds atoms because they are sensitive to atomic motions. Due to this difference, the splitting into two spatial modes with two counter-propagating controls occurs in the medium with negligible atomic motions.

### Variable beam splitting

**Figure 2:**
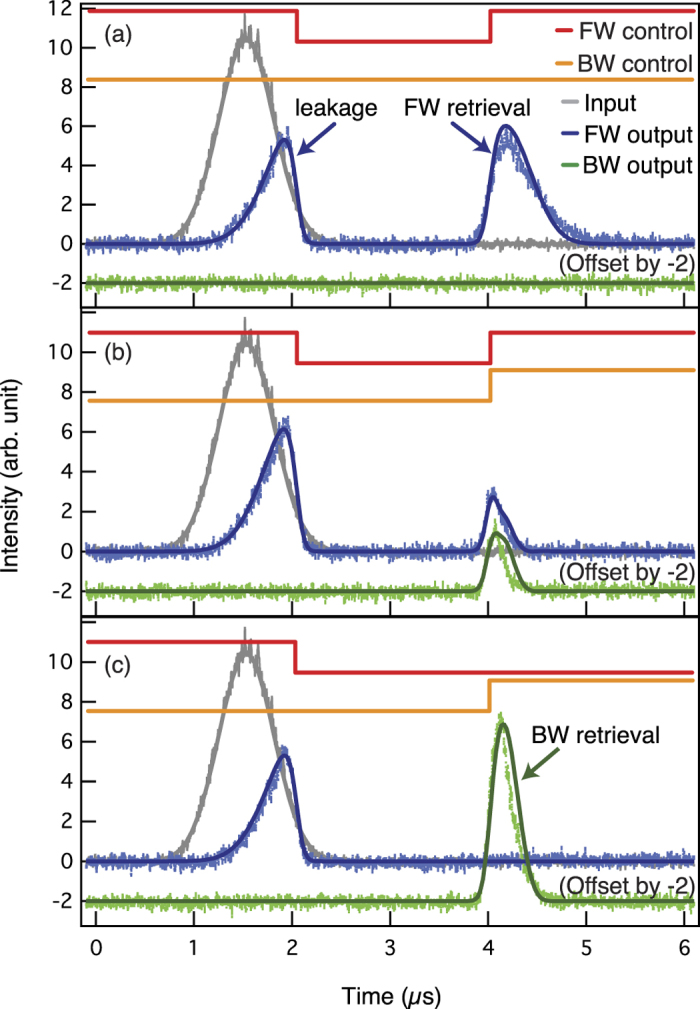
Variable beam splitting. The thick red (orange) line represents the on/off sequence for the FW (BW) control laser. The input signal represented by the grey trace is slowed down with low loss when it propagates through the EIT medium with the FW control laser. Then, it is stored into the atomic ensemble by turning off the FW control laser. The blue (green) trace shows the detected FW (BW) output. (**a**) The FW operation. The stored signal is retrieved only into the FW output by turning on the FW control. (**b**) The FW/BW beam splitting operation. The stored signal is retrieved into both the FW and BW outputs by simultaneously applying the FW and BW controls at the same Rabi frequency. (**c**) The BW operation. The stored signal is retrieved only into the BW output by turning on the BW control. In all cases, Δ = 0. The superimposed solid curves are due to numerical simulation. See text for details.

The experimental results for dynamic beam splitting are shown in Fig. 2. Here, the detuning Δ of the BW control from the |*s*〉-|*e*〉 transition is set to zero. Part of the input pulse is stored in the cold atom medium by turning off the FW control. The Rabi frequency of the FW control is Ω_FWC_ = 2*π* × (5.8 ± 0.2) MHz. The early-arriving part (indicated as leakage in Fig. 2) of the pulse leaks out from the medium without being stored before the turn-off of the FW control at 2.05 *μ*s. After a controllable delay which is set to 1.93 *μ*s, the controls are turned on for mapping the spin wave back into photonic excitation. In Fig. 2(a), only the FW control is turned on at 3.98 *μ*s with Ω_FWC_ = 2*π* × 5.7 MHz. Just as in normal EIT storage, all the stored light is retrieved into the FW output, making the cold atom ensemble to function as a coherent optical buffer or a quantum memory. For retrieving into both the FW and BW directions, i.e., the beam splitting operation, the BW control with Ω_BWC_ = 2*π* × 6.6 MHz is simultaneously turned on with the FW control as shown in Fig. 2(b). In this case, the spin wave is split into almost opposite directions in the atomic ensemble and the two counter-propagating output beams are retrieved. The medium can functions as a 50:50 beam splitter if the effective strengths of the FW and BW controls are the same. If effective Rabi frequencies of the controls are different, the medium functions as an asymmetric beam splitter in which more power is emitted in the direction of the stronger control. As shown in Fig. 2(c), it is possible that all the stored light is retrieved in the backward direction by turning on the BW control only, thus making the medium to function as a mirror integrated with a quantum memory. The pulse width of the BW output in Fig. 2(c) is narrower than that of Fig. 2(a) due to the fact that the BW control is larger, Ω_BWC_ = 2*π* × 7.7 MHz for Fig. 2(c). The Rabi frequency of the control determines the group velocity of the signal. The higher the Rabi frequency, the larger the group velocity. Therefore, the pulse in Fig. 2(c) moves with a higher group velocity in the medium, finally leaving the medium with a shorter temporal width and with a higher peak.

**Figure 3:**
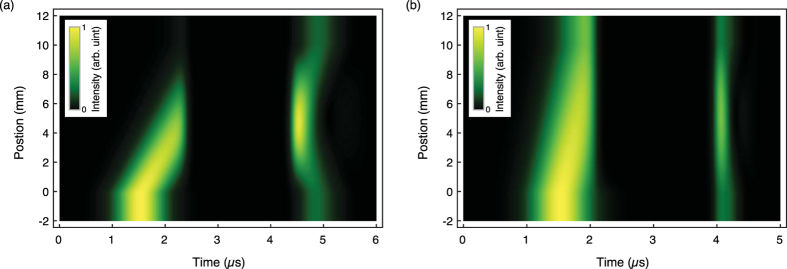
Numerical simulation of the signal propagation. The group velocity of the signal is reduced when it encounters the EIT medium located between 0 to 10 mm. (**a**) The photonic component of the signal disappears at 2.4 *μ*s when the FW control is turned off as it is mapped to the atomic spin wave excitation. By turning on the FW and BW controls at 4.4 *μ*s, the input signal stored in the EIT medium is retrieved into the FW and BW outputs. OD is set to be 100 to consider a situation with negligible signal leakage. (**b**) However, in the case of low OD of 21, the signal is not fully compressed into the medium, so the front part of it is leaving the medium before the storage. *γ*_*gs*_ = 2*π* × 3.8 kHz in both (**a**,**b**).

Our experimental results are well explained by the numerical simulation shown in the solid lines of Fig. 2. The numerical simulation is based on the Maxwell-Bloch equations for light propagation^27^. By comparing the experimental data in Fig. 2 and the results of numerical simulation using the measured values of optical depth OD and ground state dephasing rate *γ*_*gs*_, we extract the effective values of the Rabi frequencies. The two-dimensional numerical simulation in Fig. 3(a) showing the signal propagation in the medium was done by further assuming Ω_FWC_ = Ω_BWC_ = 2*π* × 7.6 MHz and OD is set to be 100 to consider a situation with negligible signal leakage.

The retrieval efficiencies of outputs in Fig. 2(a,c) are 39.7% and 38.0%, respectively. The retrieval efficiency is defined as the ratio of energy of the retrieved output (indicated as retrieval in Fig. 2) to that of the input. The input and output energies are calculated by integrating the pulse area over the time. These efficiencies can be enhanced in a medium with high OD and low *γ*_*gs*_. However, the retrieval efficiency of the sum of the FW and BW outputs in Fig. 2(b) is 20.8%, smaller than the cases with only one retrieval control. In the case where two controls are applied simultaneously, spatial higher-order Raman coherences are formed by the signals and controls. We found from the simulation, not shown here, that these coherences are not fully retrieved back to the photonic excitation at the edge of the medium, but rather partially bounces back to the medium. This is likely due to the fact that, unlike ordinary EIT, the refractive index is not unity at the medium edge when two coupling fields are applied, causing partial reflection of the coherence. One way to improve the retrieval efficiency is to apply a detuning between two control fields so that the remaining Raman coherences are well recovered back to photonic excitations. A detailed discussion of the efficiency enhancement of our beam splitting scheme is beyond the scope of this paper, and will be discussed in a separate paper. It is however instructive to mention loss mechanisms of Fig. 2 other than the Raman coherences. The main loss comes from the pulse leakage shown in Fig. 2. In the case of low OD, an input pulse is not fully compressed into the medium, making it difficult to store the full pulse width. This can be overcome by enhancing the OD as shown in Fig. 3(a). Inhomogeneous magnetic fields and atomic motions are also decreasing the efficiency by dephasing the atomic coherences.

**Figure 4:**
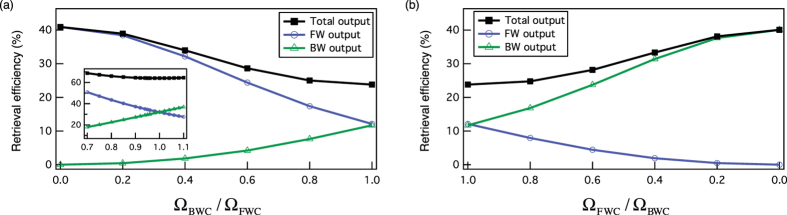
Wide tunability of the output intensities. The output intensities are tunable according to the ratio of the control Rabi frequencies. The blue (green) trace shows the retrieval efficiency of the FW (BW) output. The black trace shows the sum of the both efficiencies. Ω_BWC_ (Ω_FWC_) is varied according to the fixed Ω_FWC_ (Ω_BWC_) = 2*π* × 5.7 MHz in (**a**,**b**). This simulation is conducted with the experimental values of OD = 21 and *γ*_*gs*_ = 2*π* × 3.8 kHz. Inset in (**a**) is the extreme case of OD = 1000 and *γ*_*gs*_ = 0.

Figure 4 shows the results of numerical simulation for the variable beam splitting. It is clear that the beam splitting ratio can be varied by changing the ratio between the Rabi frequencies of the backward and forward control beams. As shown in Fig. 4, greater energy is retrieved into the FW channel than into the BW channel when the Rabi frequency of the FW control is larger than that of the BW control. The ratio of the output intensities is continuously tunable and can cover a wide range according to the ratio of the control Rabi frequencies.

### Coherence

**Figure 5:**
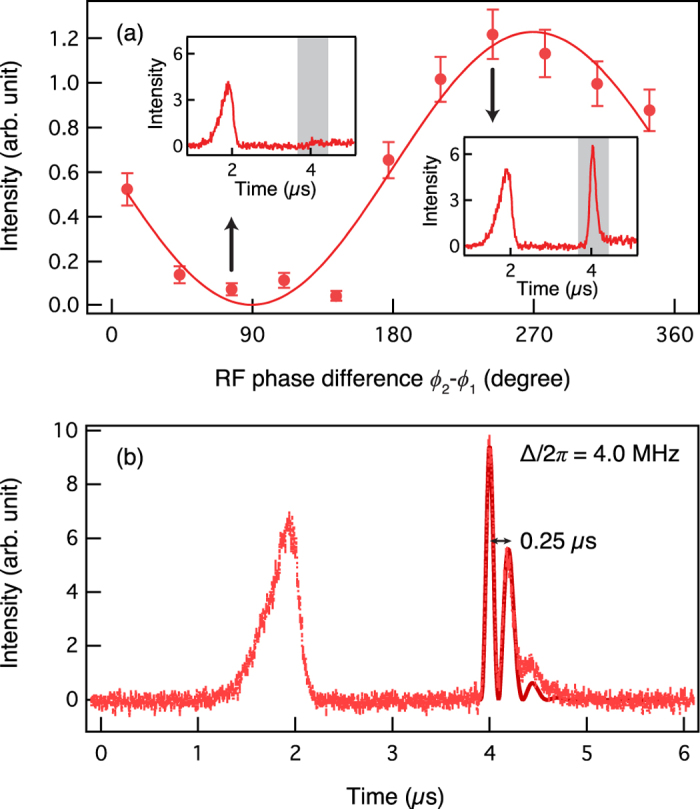
Demonstration of coherence between the two ouputs. (**a**) High visibility interference of 99.1% is observed between the two output beams in the experimental setup depicted Fig. 1. The phase difference between the two output beams can be controlled by changing the RF phase difference *ϕ*_2_ − *ϕ*_1_. Each data point is acquired by integrating the grey region of the output trace shown in the inset. The experiment is done at Δ = 0. (**b**) If the detuning Δ is non-zero for the BW control, the frequencies of the BW and FW outputs will differ, thereby causing beating between the two outputs. The solid line is the fitting curve with the oscillation period of 0.25 *μ*s.

We now examine coherence between the two output beams by superposing them at a linear optical beam splitter, forming a Mach-Zehnder interferometer in which the first beam splitter is made of a cold atom ensemble, see Fig. 1(a). If the FW and BW output beams in Fig. 2(b) are mutually coherent, high visibility interference is expected. For this experiment, the detuning Δ is set to zero so that the frequencies of the FW and BW outputs are identical. The experimental result is shown in Fig. 5(a) and it clearly shows high visibility interference between the FW and BW output beams, confirming that the beam splitting process is coherent. It is interesting to note that the phase difference between the FW and BW output beams can be varied by changing the relative phase between the two control fields. In experiment, the relative phase is controlled by the phase difference between the RF signals, *ϕ*_2_ − *ϕ*_1_, applied to the acousto-optic modulators (AOM) for generating the FW and BW controls.

As seen from equation (2), it is possible to perform a dynamic beam splitting operation in which one of the two outputs has slightly different frequency than that of the input if *ω*_C_ ≠ *ω*_FWC_. In experiment, this can be achieved by simply setting Δ ≠ 0 in Fig. 1(b). The experimental result for such a case is shown in Fig. 5(b) in which beating is observed with the period of 2*π*/Δ. This result reconfirms that the dynamic and coherent nature of the beam splitting operation based on the light storage in cold atoms.

## Discussion

We have demonstrated a coherent and dynamic beam splitter by means of an EIT-based light storage in cold atoms. Since coherent conversion between photonic and atomic spin wave excitations is at the heart of the atom-based optical beam splitter, our beam splitter scheme naturally incorporates quantum memory functions. If, instead of the conventional EIT medium, the Rydberg-EIT medium is used^28^,^29^, our beam splitter scheme can further incorporate the process of single-photon filtering from a weak laser, potentially enabling direct generation of single-photon dual-rail entanglement from a weak laser. Although the beam splitter scheme was demonstrated with a weak laser pulse, it is nevertheless applicable to the single-photon input signals^30^,^31^. Our scheme may also be used for other types of EIT media with negligible Doppler broadening^19^,^23^,^24^. Note that, in this work, we have demonstrated a 1 × 2 beam splitting device based on a cold atom medium. It is however possible to extend the scheme to *N* × *M* beam splitting, supporting *N* input and *M* outputs, using the fact that the EIT medium can be excited with multiple spin wave components and the phase matching condition allows various possible directions for light storage and retrieval. Such an *N* × *M* beam splitter based on the EIT medium may offer new possibilities for multiplexed all optical information and photonic quantum information.

Further improvements of our beam splitting work are necessary for reliable applications in quantum information processing and in all-optical signal processing. Recently, achievements of OD larger than 1,000 are reported^32^,^33^ and such a high OD medium is crucial for near unity retrieval efficiency. In addition, it is necessary to investigate the way to fully mapping atomic excitations back to photonic excitations, e.g. applying a detuning between two controls, as we suggested in our work. Moreover, integration of cold atoms into a small chip is also essential for reducing the experimental overhead in managing many optical elements, e.g., interfacing atoms with nano-photonic elements^34^,^35^.

## Methods

### Preparation of the cold atomic ensemble

87 rubidium cold atoms (<1 mK) are prepared by a MOT. After loading the neutral atomic ensemble, the trapping magnetic field is turned off and all the atoms are optically pumped into the ground state, |*g*〉 = |5*S*_1/2_, *F* = 1, *m*_*F*_ = 1〉. The other relevant atomic levels are |*e*〉 = |5*P*_1/2_, *F*′ = 2, *m*_*F*′_ = 2〉 and |*s*〉 = |5*S*_1/2_, *F* = 2, *m* = 1〉. The MOT coil is turned off to reduce the dephasing effect induced by the inhomogenous magnetic fields by the coil. The quantization axis is set by applying a weak magnetic field 0.1 G to the propagation axis of the signal field so that the polarization of the photon can be well-defined in the frame of atoms. All the lasers for preparing the cold atom medium (cooling, repumping, and optical pumping beams) are turned off not to disturb the EIT effect before the beam splitting experiment. The properties of the medium is characterized by measuring the two-level transmission and the three-level EIT transmission spectrum. The OD is measured to be 21 on the |*g*〉-|*e*〉 transition and the ground state dephasing rate is measured to be *γ*_*gs*_ = 2*π* × 3.8 kHz. OD 21 is achieved from the re-loading of the previous cycles. Our experiments is repeated every 50 ms, and the previously loaded atoms are used again for the next cycle. The atoms number is estimated to be order of 10^8^ and the MOT size is around 10 mm along the light propagation axis. The |*e*〉 state decays radiatively at rate Γ = 2*π* × 5.75 MHz. The FW control laser is first turned on, illuminating the whole atomic ensemble, after which the signal pulse is sent for the beam splitting experiment. The control beams are well collimated and the beam intensity is around 14 mW/cm^2^. The signal beam is focused to the major axis of the atomic ensemble and the beam intensity is around 25 *μ*W/cm^2^.

### Theoretical model

Our numerical simulation is based on the Maxwell-Bloch equations for light propagation, which are the first-order partial differential equations for the coupled dynamics of light and matter; equations (4–11) of Lin, Y.-W. *et al.*^27^. We assumed that the magnitudes of the wave vectors of controls and signal are the same. If the control and signal are co-propagating, they form a |*g*〉-|*s*〉 atomic coherence which is insensitive to atomic motion. The dephasing rate of this coherence is dominated by the inhomogeneous magnetic fields, which is set to be 2*π* × 3.8 kHz in our simulation. If the control and signal are counter-propagating, they form another |*g*〉-|*s*〉 atomic coherence which is sensitive to atomic motion. The dephasing rate of this coherence is dominated by the atomic motion, which is set to be 2*π* × 100 kHz in our simulation. The main parameters for characterizing the medium are OD and dephasing rates of ground state coherences. At first, these and the effective Rabi frequency of the control field are measured from a three-level EIT transmission spectrum. By fitting the measured transmission with a theoretical steady-state curve, the fitting values of the three are extracted. The result of the numerical simulation with these values almost perfectly reconstructed the measured results of light slowing, storage, retrieval, and beam splitting.

## Additional Information

**How to cite this article**: Park, K.-K. *et al.* Coherent and dynamic beam splitting based on light storage in cold atoms. *Sci. Rep.* **6**, 34279; doi: 10.1038/srep34279 (2016).
